# Visceral Leishmaniasis Associated with B-Cell Chronic Lymphocytic Leukemia: Report of a Case and Review of the Literature

**DOI:** 10.3390/life12020185

**Published:** 2022-01-27

**Authors:** Magda Zanelli, Alessandro Tafuni, Francesca Sanguedolce, Maurizio Zizzo, Andrea Palicelli, Edoardo Simonetti, Nando Scarpelli, Martina Quintini, Daniele Rosignoli, Sara Grasselli, Alberto Cavazza, Giovanni Martino, Stefano Ascani

**Affiliations:** 1 Pathology Unit, Azienda USL-IRCCS Reggio Emilia, 42123 Reggio Emilia, Italy; andrea.palicelli@ausl.re.it (A.P.); alberto.cavazza@ausl.re.it (A.C.); 2 Pathology Unit, Department of Medicine and Surgery, University of Parma, 43121 Parma, Italy; alessandro.tafuni@unipr.it; 3 Pathology Unit, Azienda Ospedaliero-Universitaria, Ospedali Riuniti di Foggia, 71122 Foggia, Italy; francesca.sanguedolce@unifg.it; 4 Surgical Oncology Unit, Azienda USL-IRCCS Reggio Emilia, 42123 Reggio Emilia, Italy; maurizio.zizzo@ausl.re.it; 5 Department of Onco- Hematology, Hospital of Spoleto, USL Umbria 2, 06049 Spoleto, Italy; simonetti87@gmail.com (E.S.); nando.scarpelli@uslumbria2.it (N.S.); 6 Hematopathology, Centro di Ricerca Emato-Oncologica—C.R.E.O., University of Perugia, 06129 Perugia, Italy; martiquintini@gmail.com (M.Q.); gio.martino@gmail.com (G.M.); 7 Department of Infectious Diseases, University of Perugia, 06129 Perugia, Italy; daniele.rosignoli@ospedale.perugia.it; 8 Infectious Diseases and Hepatology Unit, University of Parma, 43121 Parma, Italy; sara.grasselli@unipr.it; 9 Pathology Unit, Azienda Ospedaliera “Santa Maria” di Terni, University of Perugia, 05100 Terni, Italy; s.ascani@aospterni.it

**Keywords:** chronic lymphocytic leukemia, kala-azar, *Leishmania*, CD1a, pancytopenia

## Abstract

Infections often complicate the course of hematological diseases and may represent a diagnostic challenge. In particular, visceral leishmaniasis diagnosis may be missed in lymphoma patients, as lymphoma-related immunosuppression can lead to a misleadingly negative *Leishmania* serology and to atypical clinical manifestations, including the lack of fever, considered a common symptom in leishmaniasis. Herein, we report a case of visceral leishmaniasis in a patient with a long history of B-cell chronic lymphocytic leukemia presenting with increasing fatigue and diarrhea, in the absence of fever. *Leishmania* serology was negative. Bone marrow biopsy performed with the clinical suspicion of transformation to high-grade lymphoma disclosed intracytoplasmic inclusion bodies resembling *Leishmania amastigotes* within the cytoplasm of macrophages, and CD1a immunohistochemical expression helped to confirm the diagnosis of leishmaniasis. Liposomal amphotericin B was administered with complete symptom resolution. The correct identification of *Leishmania* is critical as visceral leishmaniasis represents a severe disease with an often fatal outcome, particularly in frail patients, unless promptly recognized and adequately treated. A review of the literature of visceral leishmaniasis cases occurring in B-cell chronic lymphocytic leukemia patients is performed.

## 1. Introduction

Leishmaniasis is a vector-borne parasitic disease caused by *Leishmania* spp., transmitted to humans through the bite of the sandfly *Phlebotomus*. Rodents and canines are the reservoirs of *Leishmania*, whereas humans represent the host. The main target of the parasite is the phagocytic mononuclear system [1]. Distinct species of *Leishmania* cause different clinical manifestations, ranging from self-healing lesions to life-threatening forms.

Depending on the characteristics of the different *Leishmania* species and on the host immune system, three clinical forms may occur: the cutaneous form, which is the most common; the mucocutaneous form; and visceral leishmaniasis (VL), known as kala-azar, which is the most severe and systemic form [1,2].

VL is caused by *Leishmania donovani* in Asia and Africa and *Leishmania infantum* in the Mediterranean Basin, the Middle East, Central Asia, South America, and Central America. VL is usually fatal unless promptly treated. The most common clinical manifestations are prolonged fever, splenomegaly, pancytopenia, hepatomegaly, hypergammaglobulinaemia and weight loss [3]. VL represents a common co-infection in HIV-positive individuals from endemic areas. In non-endemic countries, non-HIV-related VL is diagnosed with increasing frequency, owing to the large number of patients on treatment with immune-modulating agents for auto-immune, inflammatory and neoplastic diseases [4].

In the setting of patients with hematological neoplasms, infectious complications represent a critical issue that clinicians need to face. Hematologists should think of VL in lymphoma patients with systemic symptoms often misinterpreted as progression of the underlying lymphoma.

Herein we report a case of VL in a patient with a long history of B-cell chronic lymphocytic leukemia (B-CLL) and presenting with increasing fatigue and diarrhea in the absence of fever. Despite *Leishmania* serology negativity, bone marrow (BM) biopsy evaluation led to the identification of *Leishmania* bodies with consequent treatment, avoiding a poor outcome. Lymphoma patients affected by *Leishmania* may lack fever, and serology may be negative as a result of lymphoma-related immunosuppression; therefore, making the correct diagnosis can be particularly challenging. A review of the literature of VL cases occurring in B-CLL patients is also performed.

## 2. Case Presentation

An 80-year-old HIV-negative Italian man with a 10-year history of an otherwise indolent B-CLL presented with increasing fatigue and persistent non-bloody diarrhea not responding to antibiotics in the absence of fever. Blood tests from February 2019 to September 2020 showed a progressive decrease of neoplastic lymphocytosis (WBC from 55.170/mmc to 4.400/mmc), followed by the appearance of anemia (hemoglobin 9.2 g/dL) and thrombocytopenia (platelets 38.000/mmc). Chest radiography was negative. Abdominal ultrasonography revealed an increase of the pre-existing disease-related splenomegaly (longitudinal diameter 20 cm versus 15.5 cm) in the absence of lymphadenopathy. Toxoplasma, Epstein–Barr virus, Cytomegalovirus and *Leishmania* serologies as well as stool cultures for bacterial pathogens, ova and parasites were all negative.

**Figure 1:**
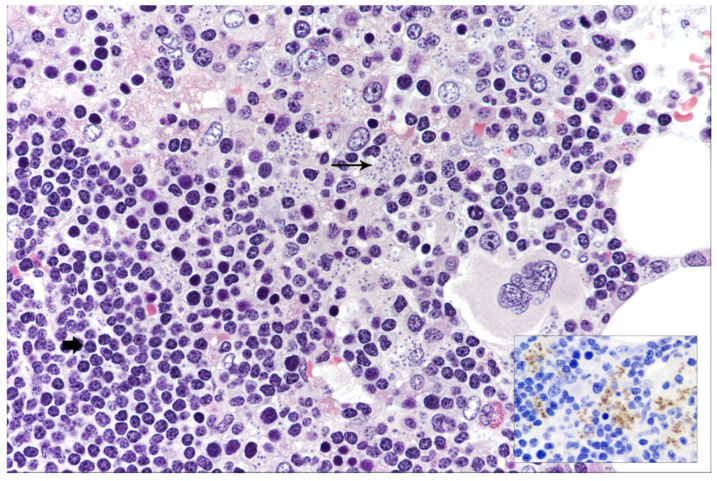
Bone marrow histology showing small-sized lymphocytes consistent with B-CLL (short and bold arrow) and tiny intracytoplasmic inclusion bodies morphologically resembling *Leishmania amastigotes* (long arrow) (hematoxylin and eosin, 400× magnification); in the inset, CD1a staining highlighting *Leishmania amastigotes* (immunostaining, 200× magnification).

In the setting of suspected transformation of the indolent lymphoproliferative disease to high-grade lymphoma, so-called Richter syndrome (RS), BM biopsy was performed. BM cellularity was markedly increased, with a diffuse, lymphoid infiltrate representing 60% of cellularity. The lymphocytes were predominantly of small size with both morphology and immunophenotype consistent with classical CLL without features of transformation (Figure 1).

Unexpectedly, at an accurate examination, macrophages with intracytoplasmic inclusion bodies morphologically resembling *Leishmania amastigotes* (Figure 1) were observed. CD1a immunohistochemical expression (Figure 1, inset) confirmed the diagnosis of VL despite the negative serology. Qualitative immunochromatographic assay on peripheral blood further supported the diagnosis. Upon demonstration of *Leishmania,* the patient was started on liposomal amphotericin B at the dose of 3 mg/kg once daily for 4 days followed by 1 dose weekly for 3 weeks. The patient obtained rapid symptom resolution, with progressive improvement of the hematological parameters (Hb: 11.8 g/dL; platelets: 85.000/mmc) and clearance of *Leishmania* at BM re-evaluation 3 months after the end of treatment. No *Leishmania* relapse occurred at 1 year of follow-up. The patient is currently receiving Ibrutinib with partial B-CLL response.

## 3. Literature Review: Methods and Results

Our literature review was performed adhering to the Preferred Reported Items for Systematic Reviews and Meta-analyses (PRISMA) guidelines. Pubmed/MEDLINE, EMBASE, Scopus, Cochrane Library, and Web of Science (Science and Social Science Citation Index) databases were used to search all related literature, using the following non-MeSH/MeSH terms: “B-cell chronic lymphocytic leukemia” and “*Leishmania*” and “visceral leishmaniasis”. The search was performed from the inception of the databases until December 2021.

The following inclusion criteria were used: (1) a diagnosis of B-CLL based on reliable morphological and immunophenotypical features according to the current criteria of the World Health Organization (WHO) Classification of Tumours of Haematopoietic and Lymphoid Tissues [5]; (2) diagnosis of VL upon demonstration of *Leishmania* bodies; and (3) retrospective, observational case-control studies, case reports and/or series, and literature reviews. The exclusion criteria were as follows: (1) studies not published in English; (2) lack of adherence to the diagnostic criteria for B-CLL according to current WHO classification [5]; and (3) lack of identification of *Leishmania* bodies. Two independent reviewers (M. Zanelli and AT) selected and identified the papers on the basis of title, abstract, keywords, and full text.

From the selected papers, the following information was collected: patient’s age, sex and HIV status; duration of B-CLL; hematological treatment performed; clinical manifestations of VL; site of detection of *Leishmania* bodies; therapy and clinical outcome. All collected results were revised by a third independent reviewer (S.A).

We identified 4 reports of VL arising in the setting of CLL [6,7,8,9], with a final number of 5 patients, including our case. In Table 1, the clinicopathological features of VL occurring in B-CLL patients are summarized.

All the patients were male, with a median age of 66 years (range: 56–80). The HIV status was negative in the 3 cases with known serological status. Four patients were Italian and one was Senegalese. No data about insect bites were reported. In all cases, a long-lasting B-CLL (from 1 to 14 years) preceded the development of VL. Data about hematological treatment were known in 4 patients. No treatment was administered to one patient. Three patients received multiple cytotoxic treatments associated with rituximab, and in 2 of these, alemtuzumab was given. Clinical manifestations included pancytopenia (5); splenomegaly (5); fever (3); fatigue (2); lymphadenopathy (2); weight loss (1); non-bloody diarrhea (1) and sweating (1). High levels of lactate dehydrogenase (LDH) and ferritin were identified in one case. Transformation to high-grade lymphoma was the clinical suspect in 3 cases, whereas one case of VL was clinically misinterpreted as hemophagocytic syndrome (HS). The diagnosis of VL was based on *Leishmania* bodies identification in BM biopsy (1); in BM aspirate and BM biopsy (1); in blood and BM aspirate (2); in lymph node and BM aspirate (1). Liposomal amphotericin B was administered with clinical remission in all cases; *Leishmania* was undetectable in the 4 cases re-evaluated at the end of treatment.

## 4. Discussion

Infections represent a significant issue in patients with hematological malignancies. Lymphoma and infections can sometimes be identified in the same histological specimen [10,11], and the emerging role of infectious agents in the pathogenesis of lymphomas is rather well-established [12,13].

B-CLL is the most prevalent type of leukemia in adults in Western countries [5]. The disease may present a prolonged and indolent course but may also become more aggressive with transformation to a high-grade lymphoma [5]. B-CLL patients are particularly predisposed to infections because of both the disease-related immunodepression and the immunosuppression (IS) caused by cytotoxic therapies and monoclonal antibodies. It has been suggested that the increasing use of immunosuppressive drugs for B-CLL treatment has determined a modification in the type of infections complicating B-CLL course from mainly bacterial infections to uncommon opportunistic infections [10].

Few cases of VL developing in lymphoma patients have been reported so far [10,11], with only five cases, including the present report, associated with B-CLL [6,7,8,9]. In all B-CLL cases developing VL, a prolonged B-CLL preceded the development of VL and, in the majority of cases, the patients had received multiple cytotoxic and immunomodulatory therapies. *Leishmania* spp are intracellular protozoa infecting the monocyte/macrophage lineage; in conditions of IS, the defense mechanisms against intracellular microorganisms are altered, leading to a severe infection that may be fatal if unrecognized and untreated.

So far, all B-CLL patients who developed VL were from regions (Italy in 4 cases and Senegal in 1 case) where leishmaniasis is endemic, and it may be hypothesized that the IS related both to long-lasting B-CLL and often multiple immunochemotherapy lines caused reactivation of a dormant infection.

In B-CLL patients, the occurrence of pancytopenia, associated with massive splenomegaly and systemic symptoms, often led the clinician to think of transformation, so-called RS. However, it is critical to remember that uncommon opportunistic infections such as leishmaniasis may closely mimic the clinical picture of RS.

Moreover, in immunocompromised individuals, the clinical manifestations of Leishmaniasis may be atypical, lacking more classical symptoms such as fever and making the diagnosis particularly challenging, as occurred in our case. For patients living in or travelling from countries where leishmaniasis is endemic, primary *Leishmania* infection or reactivation of a dormant infection should be taken into consideration, particularly in lymphoma patients under immunosuppressive treatment. Several serological tests are available for diagnostic purposes of VL.

Of note, serological tests may not be completely reliable in conditions of IS, especially in HIV-positive individuals, transplanted patients, or patients treated with drugs interfering with antibody production such as rituximab, usually displaying low or non-detectable antibodies [14,15]. Thus, seronegative cases of leishmaniasis have been observed in lymphoma patients, particularly in the setting of immunosuppressive treatment [16].

Molecular techniques, such as polymerase chain reaction (PCR) analysis, have been introduced in clinical practice [17]. PCR assay represents a sensitive and specific tool for VL diagnosis and, moreover, it is a reliable method for parasite identification at the species level, although it is not routinely employed in resource-limited countries.

However, direct observation of amastigotes in samples obtained from BM, lymph nodes or spleen still remains the gold standard for diagnosis [2]. Recently, immunostaining for CD1a has been recognized as a useful tool to identify *Leishmania* amastigotes on histological samples [18,19,20]. This technique may help when morphological features are not clear-cut and/or in case of unavailability of fresh samples; immunohistochemistry is also suitable in resource-limited settings.

## 5. Conclusions

A high index of clinical suspicion and an accurate examination of fresh and/or fixed samples is essential to recognize VL, particularly in frail and immunocompromised patients with atypical symptomatology and misleadingly negative serology. In these particular contexts, unrecognized and untreated infections may exhibit a fatal outcome and/or frequent relapses. Due to the prompt treatment with amphotericin B, all cases of VL reported in B-CLL patients so far obtained complete clinical remission with no relapse.

## Figures and Tables

**Table 1:** Literature review of B-CLL cases developing VL.

Reference Sex Age Origin HIV Status	Clinical Presentation of VL	B-CLL Duration	Hematological Therapies	Site of Leishmania Diagnosis	Leishmania Serology	Therapy/Outcome
Pitini 2012 64/M Italian HIV-	Fever pancytopenia splenomegaly	1 year	Immuno-CT: (fludarabine, cyclophosphamide, R.); (alemtuzumab)	BM aspirateand and blood	NA	Liposomal amphotericin B; resolution of symptoms; Leishmania bodies undetectable in BM
Orlandi 2014 56/M Italian HIV NA	Fatigue splenomegaly pancytopenia high ferritin high LDH (Richter transformationsuspected)	6 years	Multiple immuno-CT courses: (fludarabine, cyclophosphamide, R.); (chlorambucil) (R-benda); (alemtuzumab)	BM aspirate and BM biopsy	NA	Liposomal amphotericin B; resolution of symptoms; Leishmania bodies undetectable in BM
Nicolas 2018 73/M Italian HIV NA	Fever sweating weight loss splenomegaly lymphadenopathy pancytopenia (HS and Richter transformation suspected)	14 years	NA	Lymph node and BM aspirate	NA	Liposomal amphotericin B; resolution of symptoms
Kalmi 2020 60/M Senegalese HIV-	Fever lymphadenopathy splenomegaly pancytopenia	4 years	Immuno-CT: R-benda	Blood and BM aspirate	NA	Liposomal amphotericin B; resolution of symptoms; clearance of Leishmania DNA from blood
Present case 80/M Italian HIV-	Fatigue non-bloody diarrhea pancytopenia splenomegaly (Richter transformation suspected)	10 years	No therapy	BM biopsy	Negative	Liposomal amphotericin B; resolution of symptoms; Leishmania bodies undetectable in BM

Note: BM: bone marrow; B-CLL: B-cell chronic lymphocytic leukemia; CT: chemotherapy; HIV: human immunodeficiency virus; HS: hemophagocytic syndrome; LDH: lactate dehydrogenase; M: male; NA: not available; R: rituximab; VL: visceral leishmaniasis.

## Data Availability

The data presented in this study are available on request from the corresponding author.
